# Predicting Procedure Time in Pediatric Dental Rehabilitation Under General Anesthesia: The Role of Preoperative Factors and Age‐Based Models

**DOI:** 10.1111/pan.70087

**Published:** 2025-11-20

**Authors:** R. J. Banchs, K. Barawi, B. A. Banchs, E. Kratunova

**Affiliations:** ^1^ Department of Anesthesiology College of Medicine, University of Illinois Chicago Chicago Illinois USA; ^2^ Department of Oral and Maxillofacial Surgery College of Dentistry, University of Illinois Chicago Chicago Illinois USA; ^3^ Department of Integrative Biology University of Illinois at Urbana Champaign Urbana Illinois USA; ^4^ Department of Pediatric Dentistry College of Dentistry, University of Illinois Chicago Chicago Illinois USA

## Abstract

**Background:**

Dental rehabilitation under general anesthesia (GA) is often required for children who are unable to cooperate during standard dental procedures. Accurately estimating the duration of these cases is challenging, particularly when preoperative X‐rays are unavailable. Efficient scheduling and optimal operating room (OR) utilization rely on precise time predictions; however, existing predictive models, including EPIC's analytics, frequently overlook patient‐ and case‐specific factors, resulting in suboptimal OR efficiency.

**Aims:**

This study aimed to identify preoperative, patient‐specific factors that influence the duration of pediatric dental rehabilitation under GA and to develop an age‐based predictive equation to improve procedure time estimation.

**Methods:**

A retrospective review was conducted on 255 dental rehabilitation cases performed under general anesthesia (GA) between January 2022 and December 2023. Collected data included patient demographics, treatment details, availability of radiographs, and operating room (OR) time metrics. Statistical analysis was performed to assess the influence of preoperative factors on procedure duration. An age‐based fitted equation was developed, and its predictive accuracy compared with that of EPIC's analytics system.

**Results:**

Age was the strongest patient‐specific predictor of procedure duration (*p* < 0.001, *R* ^2^ = 50.73%), correlating with both dentition type and the extent of dental restoration required. The age‐based fitted equation substantially outperformed EPIC's analytics, particularly in the 3–5 and 13–18 age groups, improving prediction accuracy by 42% and 114%, respectively. The fitted equation was Y = 84–4.5X + 0.6X^2^, where Y represents procedure time and X represents age. Other patient‐specific variables, including weight, BMI, and ASA classification, demonstrated minimal influence.

**Conclusions:**

Developing an age‐specific fitted equation based on site‐specific operating room (OR) data improves procedure time prediction for pediatric dental rehabilitation under GA. This model supports more precise scheduling, better resource allocation, and improved patient access to care, providing a valuable framework for efficiency in the OR.

## 1 Background

Dental rehabilitation under general anesthesia (GA) in the operating room (OR) is often required for pediatric patients and adults with complex medical or behavioral conditions that make conventional dental treatment impractical [1]. These patients commonly face substantial dental disease burdens and disparities in access to care, particularly within underserved communities [2]. Efficient OR utilization is therefore critical to minimizing wait times and ensuring timely treatment for urgent cases.

Reliable prediction of procedure duration is essential for optimizing OR scheduling and resource allocation [3]. Existing predictive systems, such as EPIC's analytics platform, estimate case length primarily based on procedure type and the provider's historical averages. While useful as a baseline, these models often lack precision because they fail to incorporate patient‐ and case‐specific variables, such as age, treatment complexity, and the number of teeth requiring intervention. As a result, simple cases involving a few extractions may be scheduled with the same estimated duration as more complex cases requiring multiple restorations or pulpal therapies.

Prior studies have underscored these limitations, emphasizing the need for more refined predictive approaches tailored to dental rehabilitation [4, 5, 6]. By integrating key patient and procedural factors—particularly age and the extent of required treatment—predictive accuracy can be improved, enhancing scheduling efficiency and reducing delays in care delivery.

The present study sought to identify preoperative factors influencing procedure duration and to develop a fitted equation to improve time prediction for pediatric dental rehabilitation under GA. The resulting model aims to support quality improvement initiatives by increasing the precision of case time estimates and optimizing OR scheduling and resource management.

## 2 Materials and Methods

This retrospective cross‐sectional study was conducted at the University of Illinois Chicago Hospital, a tertiary care teaching institution and the largest public healthcare provider in Illinois. It included cases performed in the main OR between January 1, 2022, and December 31, 2023, involving patients aged 3–18 years referred by the College of Dentistry for comprehensive dental rehabilitation under GA. Institutional Review Board approval was obtained as part of a QI initiative.

Data were collected from the EPIC electronic health record system (EPIC Systems, Verona, WI, USA). Exclusion criteria included non‐dental procedures, combined surgeries, and cases performed exclusively by oral surgeons. 15% of cases were excluded due to incomplete or unreliable records to ensure high data integrity. Each case was reviewed by designated investigators: anesthesia‐related data were collected by the anesthesia team, while dental data were obtained by the dental team, leveraging each group's domain expertise to maintain accuracy.

Recorded variables included patient demographics (age, weight, BMI, and ASA classification). Dental data included dentition type (primary, mixed, or permanent), total number of teeth treated, and a detailed count of teeth extracted, restored with stainless steel crowns (SSCs) or composite resins, treated with sealants, or managed with endodontic procedures such as pulpotomies. The number and type of radiographs obtained during the procedure were also recorded. All treatments were performed by pediatric dentistry residents under the supervision of attending faculty. Provider‐related variables, including years of experience for both anesthesia and dental teams, were collected to assess their potential influence on procedure duration and outcomes.

Time‐related metrics were extracted from operative and anesthesia records to capture the entire procedural workflow. Key timestamps included patient entry into the OR, anesthesia induction, readiness for the procedure, procedure start and end, and patient exit. Additional timestamps from the post‐anesthesia care unit (PACU), including entry and anesthesia stop, were used to calculate induction, procedure, emergence, total anesthesia, total OR, and turnover times. This comprehensive dataset allowed for the integration of patient‐, treatment‐, and provider‐specific variables, serving as the foundation for predictive modeling and OR optimization.

EPIC's internal predictive system estimated procedure times by averaging the durations of the last seven cases performed by each provider. For this study, “accuracy” was defined as a predicted time falling within ±15% of the actual recorded duration, aligning with institutional benchmarks for scheduling efficiency. Predictions outside this range were categorized as overestimations or underestimations. Predicted times from EPIC were compared with actual case durations to evaluate model performance.

Data analysis was performed using Minitab statistical software (Minitab LLC, State College, PA, USA). Descriptive statistics summarized patient demographics, treatment details, radiographic use, provider experience, and time intervals. Factors influencing procedure time were grouped into: Patient‐related variables (age, weight, BMI, ASA classification, dentition type); Treatment‐related variables (number of teeth treated, extractions, restorations with SSC, composite, or sealant, and endodontic treatments.); Provider‐related variables (years of experience and resident training level).

Statistical analyses included *t*‐tests for comparing means, ANOVA for multiple group comparisons, Tukey's HSD for post hoc differences, the Kruskal–Wallis test for median comparisons, and simple and multiple regression analyses to identify predictors of procedure duration. Among patient‐related variables, age and dentition type emerged as key predictors. A quadratic relationship between age and procedure time was validated through regression modeling to generate a fitted equation. Statistical significance was set at *p* < 0.05.

## 3 Results

The initial dataset consisted of 300 cases, which were reviewed for eligibility based on the predefined criteria. A total of 45 cases (15%) were excluded, 27 cases (9%) for incomplete data, and 18 cases (6%) for unreliable records. This process resulted in a final sample of 255 cases for analysis. These cases included patients aged 3–18 years, who were categorized into three age groups based on developmental stages of dentition that reflect the differing treatment needs and procedural complexities associated with each stage of dental development (Table 1). The BMI of participants in Group 1 and Group 2 was similar (Table 2) whereas it was significantly lower than the BMI in Group 3 (*p* < 0.002, 95% CI: 18, 26). ASA classifications were as follows: 23.9% of participants were ASA I (*n* = 61), 60.39% were ASA II (*n* = 154), and 15.69% were ASA III (*n* = 40). All groups had a higher number of patients in the ASA class II: Group 1 (38% ASA I, 52% ASA II, 10% ASA III); Group 2 (21% ASA I, 65% ASA II, 14% ASA III); Group 3 (6% ASA I, 58% ASA II, 36% ASA III). No significant differences were found between Group 1 and Group 2 regarding the total number of teeth treated, rehabilitated, or extracted (Table 3). However, Group 3 showed a significant difference in the total number of teeth treated, rehabilitated, and extracted when compared to the other two groups (*p* < 0.001). The number of SSCs was highest in Group 1, while the number of composite resin restorations and sealants was highest in Group 3 (*p* < 0.001). A total of 38 patients (15%) required a pulpotomy. Group 1 had a higher percentage of pulpotomies compared to Group 2 (1 pulpotomy: 19% vs. 8%; 2 pulpotomies: 5.48% vs. 2.68%; 3 pulpotomies: 2.74% vs. 0.67%; 4 pulpotomies: 1.37% vs. 0%). No patients in Group 3 received a pulpotomy.

**TABLE 1:** Percentage of primary, mixed, and permanent teeth for each age group.

Age category	Dentition type		
	Primary	Mixed	Permanent
Group 1 (3–5 years)	83.56%	16.44%	0%
Group 2 (6–12 years)	3.36%	85.91%	10.74%
Group 3 (13–18 years)	0%	6.06%	93.94

**TABLE 2:** Mean and standard deviation for patient age, weight, and BMI for each age group.

Variable	Age group		
	Group 1	Group 2	Group 3
Age (in years)	4.2 (0.7)	7.9 (1.7)	14.7 (1.4)
Weight (in kg)	22.68 (7.5)	33.88 (15.45)	59.94 (20.26)
BMI (m^2^/kg)	19.07 (4.1)	20.09 (6.3)	23.77 (6.55)

**TABLE 3:** Median number of teeth treated, rehabilitated, extracted and type of treatment for each age group.

Variable	Age group		
	Group 1	Group 2	Group 3
Total number of teeth treated	12	12	16
Number of teeth rehabilitated	8	8	15
Number of teeth extracted	4	4	1
Stainless steel crowns	4	3	0
Composite resin restorations	2	2	8
Dental sealants	0	3	4

*Note:* The “total number of teeth treated” refers to the sum of teeth rehabilitated, and teeth extracted.

All surgical teams had a similar composition and level of experience. Each team consisted of one surgical attending (average 7 years of experience) and two pediatric dental residents (one senior and one junior). All anesthesia teams included one attending anesthesiologist (average 8 years of experience) and one junior or senior resident. No significant differences (*p* = 0.236) were observed in average induction, emergence, or total anesthesia time across the groups (Table 4). The average total anesthesia time accounted for 27%, 25%, and 16% of the total OR time for Groups 1, 2, and 3, respectively. The average procedure time for Groups 1 and 2 did not differ significantly (*p* < 0.001); however, procedure time for Group 3 was significantly longer (*p* < 0.001). The average procedure time for ASA I patients, ASA II patients, and ASA III patients was not statistically significant (*p* = 0.060). When comparing actual procedure times to EPIC's predicted times, 32.94% of procedure times were accurately predicted, while 67.07% were not (32.94% overestimated and 34.13% underestimated). In Groups 1 and 2, the percentages of accurately predicted procedure times were similar (32.88% vs. 35.62%), as were the percentages of inaccurately predicted times (67.12% vs. 64.38%). In contrast, 78.79% of procedure times in Group 3 were not accurately predicted, with 66.67% being underestimated. The number of primary teeth treated did not significantly affect the accuracy of the procedure time predictions (*p* = 0.190). However, the number of permanent teeth treated significantly contributed to underestimating procedure time (*p* < 0.001). Regression analysis showed that patient factors had a variable impact on procedure time. Age was a significant factor, with increasing age correlating with longer procedure times (*p* < 0.001, *R* ^2^ = 50.73%). In contrast, patient weight, BMI, and ASA classification had minimal influence on procedure time, as indicated by low *R* ^2^ values (12.45%, 0.86%, and 3.42%, respectively). The relationship between procedure time (Y) and age (X) followed a quadratic model: y = 84.40–4.5X + 0.6X^2^. Dental treatment factors also influenced procedure time. Although various procedures were evaluated, these factors alone were insufficient to predict procedure time without preoperative radiographs. Procedure time was directly proportional to the total number of teeth treated (*p* < 0.001, *R* ^2^ = 31.25%). The number of rehabilitated teeth had a stronger impact on procedure time than the number of extracted teeth (*R* ^2^ = 35.49% vs. 0.92%). Furthermore, the number of permanent teeth treated was a better predictor of procedure time than the number of primary teeth (*R* ^2^ = 47.22% vs. 26.44%). The type of dental procedure also significantly influenced procedure time (*p* < 0.001, *R* ^2^ = 49.34%). Among the treatment types, composite resin restorations had the greatest effect on procedure time variability (*R* ^2^ = 42.17%), when compared to SSCs (*R* ^2^ = 8.90%) and dental sealants (*R* ^2^ = 4.24%). Provider factors, such as the experience level of the surgical team, did not significantly influence procedure time. Multiple regression analysis showed that combining patient factors did not significantly improve the ability to predict procedure time. However, combining age with the number of permanent teeth requiring composite treatment provided the most effective prediction of procedure time (*R* ^2^ = 51%). The quadratic equation Y = 84–4.5X + 0.6X^2^, representing the relationship between age and procedure duration in our OR, was applied to calculate predicted procedure times. These predictions were then compared with both EPIC's predicted times and the actual recorded durations to assess accuracy. The analysis revealed that while the quadratic model and EPIC's analytics demonstrated comparable accuracy in Group 2 (35.62% vs. 35.64%, respectively), the quadratic model substantially outperformed EPIC in other groups, improving prediction accuracy by 42% in Group 1 (from 32.88% to 46.58%) and by 114% in Group 3.

**TABLE 4:** Mean of different time intervals.

Variable in minutes	Age group		
	Group 1	Group 2	Group 3
Induction time	18 (15.8, 20.2)	16.2 (15, 17.40)	18.21 (14.9, 21.5)
Emergence time	15.44 (14.1, 16.7)	17.17 (15.7, 18.6)	15.76 (13.1, 18.4)
Total anesthesia time	33 (30.3, 35.6)	33.38 (31.4, 35.4)	33.97 (29.4, 38.5)
Procedure time	75.90 (70.7, 81)	83.4 (77.8, 89)	148.47 (128.6, 168.3)
Total OR time	124 (118.6, 129.7)	133.28 (127.5, 139.1)	205.3 (184.8, 225.7)

## 4 Discussion

Comprehensive dental rehabilitation under GA enables the treatment of all necessary dental procedures in a single session [2, 7], ensuring optimal care for pediatric patients. Age was the most significant predictor of procedure duration, with a quadratic relationship with procedure time. This model significantly outperformed EPIC's analytics, particularly for younger children and adolescents, with prediction improvements of 42% and 114%, respectively. To analyze procedural variability and identify treatment patterns, patients were stratified into age groups based on developmental stages of dentition. This stratification was necessary for understanding how factors like the number of composite resin restorations or SSCs varied across age ranges, directly influencing procedure duration. Age was incorporated as a continuous variable in the predictive model to ensure broader applicability, avoiding the limitations of group‐specific models. The use of percentage accuracy (±15%) in this study aligns with institutional benchmarks for evaluating predictive model performance. The significant impact of age on procedure duration can be attributed to the complexity and number of dental procedures required at different developmental stages. Younger children often present with extensive dental caries in primary teeth, requiring treatments like extractions and SSCs, which are relatively quicker [8]. In contrast, older children and adolescents have permanent teeth that often need more complex and time‐consuming procedures, such as composite resin restorations and endodontic therapies [9]. Composite resin restorations, in particular, were found to significantly influence procedure time variability (*R* ^2^ = 42.17%) because they involve a technique‐sensitive process, including precise application and light curing, which contributes to extended procedure durations compared to other dental treatments. The prediction accuracy for Group 2 was low in both EPIC and the age‐based fitted equation models given the heterogeneity of primary and permanent teeth found in this age group. Contrary to findings in other surgical specialties where patient weight, BMI and ASA classification are significant predictors of procedure duration, this study found that these factors had minimal impact on the duration of dental rehabilitation procedures under GA in pediatric patients [10, 11]. This suggests that in pediatric dentistry, the complexity and type of dental procedures, influenced by age and dentition, are more critical determinants of procedure time than general health status or anthropometric measures. The lack of significant influence from provider‐related factors, such as the experience level of the surgical and anesthesia teams, indicated a standardized level of care in the teaching hospital setting where the study was conducted. All procedures were supervised by experienced attending faculty, which may have reduced variability due to provider skill levels. Previous studies have shown mixed results regarding the impact of provider experience on procedure duration, with some suggesting that team familiarity and experience can reduce operative times [12], while others found no significant effect [13]. An important consideration is the role of preoperative radiographs in treatment planning and procedure duration. In cases where preoperative radiographs are unavailable, additional time is required intraoperatively to obtain and interpret imaging, develop a comprehensive treatment plan, and perform all necessary procedures in a single session. This can extend procedure times and introduce variability that is difficult to predict [14, 15]. Efforts on taking preoperative radiographs, when feasible, could enhance the accuracy of treatment planning and procedure time predictions [14, 15].

Holmes et al. [16] discussed how electronic health records like EPIC present challenges for clinical care and research due to their lack of granularity in procedure‐specific data. Stepaniak et al. [17] found that models predicting surgical times based solely on historical averages often result in discrepancies, especially in cases with variable complexity. A study by Aljaffary and colleagues evaluating surgery duration accuracy in 97 397 cases found significant inefficiencies due to over‐ and underestimations of procedure times, recommending the integration of patient‐specific factors into predictive tools to enhance scheduling precision [18].

The development of the age‐based fitted equation using site‐specific data represents a practical and effective approach to improving procedure time predictions. By incorporating patient age and specific dental treatment factors into predictive models, hospitals can enhance scheduling accuracy and optimize OR utilization. This is particularly important where efficient use of limited OR resources can reduce patient waiting times and improve access to essential dental care [19]. Integrating the predictive model into electronic health record systems and scheduling software could facilitate its adoption in clinical practice. Collaboration with software developers to enhance existing predictive analytics tools, such as EPIC's system, by incorporating variables like age and specific dental procedures, could extend the benefits to a broader range of institutions [20]. This study also highlights the limitations of current predictive analytics in accurately estimating procedure times for pediatric dental rehabilitation under GA. EPIC's system, which bases predictions on the average duration of the last seven procedures performed by each surgeon, does not account for patient‐specific factors such as age and treatment complexity. This can lead to significant underestimations or overestimations of procedure times [21]. Overestimations of procedure time can lead to underutilized OR blocks, while underestimations can result in overtime costs and scheduling conflicts [22]. There are a number of limitations that warrant consideration. The retrospective design may have introduced selection bias, although efforts were made to include all eligible cases within the study period. The data were collected from a single tertiary care teaching institution, which may limit the generalizability of the findings to other settings with different patient populations or practice patterns. Additionally, the study did not account for intraoperative factors that could influence procedure duration, such as unexpected complications, equipment availability, or variations in anesthesia techniques. Exploring the integration of the model into electronic scheduling systems and evaluating its impact on OR efficiency, patient wait times, and overall access to care would also have been beneficial. Future studies examining the cost‐effectiveness of implementing such predictive models could support their adoption in healthcare institutions.

## 5 Conclusions

Age was identified as a significant predictor of procedure duration in dental rehabilitation under GA, with older pediatric patients requiring longer operative times. Other patient factors, including weight, BMI, and ASA classification, had minimal influence on procedure length. Dental treatment variables, such as the total number of teeth treated and the specific types of procedures performed, also affected duration but could only enhance prediction accuracy when preoperative radiographs were available. Developing an age‐based fitted equation using site‐specific OR data markedly improved predictive precision. The model derived in this study (Y = 84–4.5X + 0.6X^2^) outperformed EPIC's analytics, particularly among younger and older age groups. The accuracy of EPIC's predictive system could be further strengthened by incorporating patient age and, when available, the number of permanent teeth requiring composite resin restorations.

## Data Availability

The data that support the findings of this study are available on request from the corresponding author. The data are not publicly available due to privacy or ethical restrictions.
